# Incidence, microbiology, and patient characteristics of skin and soft-tissue infections in a U.S. population: a retrospective population-based study

**DOI:** 10.1186/1471-2334-13-252

**Published:** 2013-05-30

**Authors:** Gary Thomas Ray, Jose Antonio Suaya, Roger Baxter

**Affiliations:** 1 Division of Research, Kaiser Permanente Medical Care Program, Northern California Region, 2000 Broadway, Oakland, CA 94612, USA; 2 Health Outcomes, North America Vaccine Development, GlaxoSmithKline Vaccines, 16th Street, 200 N, Philadelphia, PA, 19102, USA; 3 Kaiser Permanente Vaccine Study Center and The Permanente Medical Group, 1 Kaiser Plaza, Oakland, CA, 94612, USA

**Keywords:** Skin and soft-tissue infections, Methicillin-resistant staphylococcus aureus, Incidence, Epidemiology

## Abstract

**Background:**

Skin and soft tissue infections (SSTIs) are commonly occurring infections with wide-ranging clinical manifestations, from mild to life-threatening. There are few population-based studies of SSTIs in the period after the rapid increase in community-acquired methicillin-resistant *Staphyloccus aureus* (MRSA).

**Methods:**

We used electronic databases to describe the incidence, microbiology, and patient characteristics of clinically-diagnosed skin and soft tissue infections (SSTIs) among members of a Northern California integrated health plan. We identified demographic risk factors associated with SSTIs and MRSA infection.

**Results:**

During the three-year study period from 2009 to 2011, 376,262 individuals experienced 471,550 SSTI episodes, of which 23% were cultured. Among cultured episodes, 54% were pathogen-positive. *Staphylococcus aureus* (*S*. *aureus*) was isolated in 81% of pathogen-positive specimens, of which nearly half (46%) were MRSA. The rate of clinically-diagnosed SSTIs in this population was 496 per 10,000 person-years. After adjusting for age group, gender, race/ethnicity and diabetes, Asians and Hispanics were at reduced risk of SSTIs compared to whites, while diabetics were at substantially higher risk compared to non-diabetics. There were strong age group by race/ethnicity interactions, with African Americans aged 18 to <50 years being disproportionately at risk for SSTIs compared to persons in that age group belonging to other race/ethnicity groups. Compared to Whites, *S*. *aureus* isolates of African-Americans and Hispanics were more likely to be MRSA (Odds Ratio (OR): 1.79, Confidence Interval (CI): 1.67 to 1.92, and, OR: 1.24, CI: 1.18 to 1.31, respectively), while isolates from Asians were less likely to be MRSA (OR: 0.73, CI: 0.68 to 0.78).

**Conclusions:**

SSTIs represent a significant burden to the health care system. The majority of culture-positive SSTIs were caused by *S*. *aureus*, and almost half of the *S*. *aureus* SSTIs were methicillin-resistant. The reasons for African-Americans having a higher likelihood, and Asians a lower likelihood, for their *S*. *aureus* isolates to be methicillin-resistant, should be further investigated.

## Background

Skin and soft tissue infections (SSTIs) are common clinical conditions ranging from mild to life-threatening [1,2]. Because many episodes of SSTIs are not cultured, the most common causes of SSTIs in general remain uncertain, although *Staphylococcus aureus* (*S*. *aureus*) and beta-hemolytic streptococci (BHS) are often suggested as being the most important [3-6]. Among culture-confirmed SSTIs in the United States, the most common cause is *S*. *aureus*, although *Pseudomonas aeruginosa*, *Enterococcus spp*., *Escherichia coli*, and BHS have also been identified as important causes of some types of SSTIs [1,7-11].

Infections due to *S*. *aureus* present a significant health problem in the United States [12-14], and treatment of these infections has become more difficult in the last decade due to the emergence and rapid spread of methicillin-resistant *S*. *aureus* (MRSA) [13-15]. Between 1995 and 2005 there was a dramatic increase in community-associated MRSA (CA-MRSA), and this increase was most commonly associated with SSTIs [16-22]. Studies reported increasing numbers of emergency department visits and hospitalizations for SSTIs [23-25], contemporaneous with the emergence of CA-MRSA. A study from England reported a nearly two-fold increase in cellulitis-related hospitalizations in children from 1997 and 2006 [26]. However, there are some indications that the rate of MRSA infections has stabilized or declined in the last few years [27-30].

In a recently published report of trends in *S*. *aureus* among a Northern California population, we found that nearly 60% of culture-confirmed *S*. *aureus* infections were associated with a diagnosis of SSTI, and that the number of positive *S*. *aureus* cultures identified in this population was significantly higher in recent years (2006 to 2009) than in earlier years (1998–2001) [29]. However, because many SSTIs do not receive microbiological testing, analyses restricted to culture-confirmed infections do not fully address issues of disease burden. There are very few population-based estimates of the incidence of SSTI episodes requiring medical care, especially ones including years after the rapid rise in CA-MRSA, and which include non-cultured SSTIs and SSTIs treated in outpatient settings [26,31]. Given the paucity of large, population-based studies of SSTIs in all settings, with and without cultures, we conducted this study in a general membership of a large, integrated health plan, with the objective of determining the annual incidence of SSTIs, the rate and results of microbiological testing associated with SSTIs, and demographic and clinical risk factors for SSTIs, and SSTIs with culture-confirmed *S*. *aureus* and MRSA.

## Methods

### Setting

Kaiser Permanente of Northern California (KPNC) is a nonprofit, integrated health care delivery system providing care to over 3 million members. The member population reflects the general population in the Northern California region, although, as an insured population, it under-represents persons with very low levels of education and income [32]. Clinical diagnostic and laboratory results data from hospital discharges and ambulatory settings, including emergency departments, are archived in electronic medical record and administrative databases. KPNC databases contain individual patient records and are readily linked using the patient’s unique medical record number which remains with the patient for life. In this retrospective, observational, study, clinical decisions regarding diagnosing and testing reflect usual care by KPNC providers [29]. The study was approved by the KPNC Institutional Review Board.

### Definition of SSTI episodes

Using electronic databases, we identified all clinic visits, Emergency Department visits, and hospital-based encounters occurring between January 1, 2009 and December 31, 2011, with a diagnosis (International Classification of Diseases, 9th Revision, Clinical Modification [ICD9-CM]) relating to SSTIs. We selected this three-year period in order to reflect current practice patterns, to reflect a period when MRSA incidence appears to have stabilized [29], and because it was the most recent period for which we had complete diagnostic and laboratory data available for this population. For hospital-based encounters, we only included SSTIs that were listed as the primary diagnosis, thus likely excluding hospital-onset cases – although some cases with a second co-primary diagnoses such as sepsis will also have been excluded. SSTIs were defined by ICD9-CM code as the following: Cellulitis and abscess, comprising abscess of anal and rectal region (566×), abscess of breast (6751×, 6752×), cellulitis and abscess of finger and toe (681×), other cellulitis and abscess (682×), and pilonidal cyst with abscess (6850); Carbuncle and furuncle (680×); Impetigo (684); Other infections of skin and subcutaneous tissue (686×); Folliculitis (7048×); and Other skin and soft tissue infections, comprising erysipelas (035), other mastitis (6752×), acute lymphadenitis (683), hydradenitis (70583), and necrotizing fasciitis (72886). These codes include most of those used by Edelsberg et al [25]. in their analysis of hospitalizations caused by SSTIs, plus erysipelas, abscess of breast, nonpurulent mastitis, folliculitis and hydradenitis. Because our focus was on acute, community-onset SSTIs, and not on nosocomial or chronic SSTIs, we did not include in these analyses chronic/decubitus ulcer, surgical site infection, post-operative wound infection, infection due to device, gangrene, or amputation stump infection.

SSTI episodes were defined as beginning on the date of the first SSTI diagnosis and ending with the last SSTI or complication diagnosis, not followed by another SSTI or complication diagnosis within 42 days. A six week period was considered short enough as to be unlikely to include multiple different episodes, but long enough to extend beyond the time that a patient might be asked to return for a follow-up visit. The start of an episode was defined solely by one of the diagnoses noted above. However, receipt of one or more complications diagnoses could prolong the episode. The following ICD-9-CM codes were considered potential SSTI complications: 038 (sepsis/septicemia), 04082 (toxic shock syndrome), 7280 (myositis), 730× (osteomyelitis), 7854 (gangrene), 78552 (septic shock), 7970× (bacteremia), 99590–99592 (systemic inflammatory response syndrome.) We retained only those episodes where the patient was a KPNC member at the start of the episode.

### Patient demographics and block group income

Patient age and gender were extracted from KPNC membership databases. Although race/ethnicity is not systematically captured for all members, the race/ethnicity of a majority of patients (about 94%) was identified via a search of electronic medical records, membership databases and other administrative databases. Patients were classified as Hispanic if their ethnicity was identified as “Hispanic” in any of these sources. For patients who did not have race/ethnicity recorded in administrative databases, we imputed race/ethnicity using the Bayesian Improved Surname and Geocoding algorithm, which uses census data and surname to impute the probabilities that the patient belonged to each of six different racial/ethnic groups [33].

Patient addresses were extracted from membership databases and were used to determine the patient’s census block group. Using the American Community Survey 2006–2010 Summary File [34], we determined the median block group income for each patient, and classified their SSTIs based on the quintile of the block group median income. Persons whose address information did not map to a valid census block group were classified into a sixth category “unknown”.

### Diabetes status as comorbidity of interest

Because persons with diabetes are widely considered to be at increased risk of SSTIs [35-40], we classified SSTI episodes by the diabetes status of the patient. An SSTI episode was considered to be for a patient with diabetes if the patient was on the KPNC Diabetes Registry and was considered to have diabetes prior to the start of the episode. The KPNC Diabetes Registry was established in 1993 to monitor the quality of care and health outcomes for plan members with diabetes and is updated annually by identifying all plan members with diabetes from automated databases of pharmacy data, laboratory data, hospitalization records, and outpatient diagnoses [41-43].

### Microbiology of SSTIs

Microbiology test results were extracted from KPNC’s Laboratory Utilization and Reporting System. We retained for analysis all blood, body fluid, tissue, and other miscellaneous bacterial specimens (such as those taken from abscesses, pustules, boils, wounds, etc.) obtained from patients with SSTIs in the period from 7 days prior to the start of their SSTI episode to 7 days after the end of their SSTI episode. These tests were considered to have been performed to determine SSTI etiology. We excluded respiratory, cerebrospinal fluid and urine specimens. For those SSTI episodes where a microbiology test was ordered, we defined the “index specimen” as being the specimen from which the first clinically-relevant organism was isolated, or the first specimen obtained during the relevant time period if no organism was isolated. The isolate from the index specimen was defined as the “index isolate”, and we considered this organism to be the cause of the infection. Episodes could be associated with more than one type of pathogen if multiple pathogens were identified from the index specimen.

Organisms were considered clinically-relevant pathogens only if the laboratory performed antibiotic susceptibility testing on the isolate, or if it was one of the following organisms which do not routinely receive antibiotic susceptibility testing: BHS, *Streptococcus pneumoniae*, *Bacteroides spp*., *Fusobacterium spp*., *Haemophilus spp*., *Pasteurella spp*., *Peptococcus*, *Porphyromonas spp*., *Prevotella spp*., *Peptostreptococcus spp*., and *S*. *aureus*. The following organisms were considered contaminants and were not counted as clinically-relevant pathogens: coagulase negative staphylococcus, *Ochrobactrum*, *Corynebacterium*, *Micrococcus spp*., *Bacillus spp*., gram positive cocci, gram positive rods, and streptococcus species other than BHS, *S*. *pneumoniae*, *Streptococcus milleri*, *Streptococcus anginosus*, *Streptococcus constellatus*, and *Streptococcus intermedius*. In addition, some uncommon organisms that could not be definitively speciated by the laboratory were also excluded.

### Crude incidence rates and predictors of clinically-diagnoses SSTIs

The denominator used for calculating incidence rates was KPNC member years. We first identified all persons who were members of KPNC at any time between January 1, 2009 and December 31, 2011. KPNC membership systems track membership on a monthly basis and persons could enter and leave the health plan throughout this time. We summarized member months into strata by calendar year (2009, 2010 and 2011), age (in one-year increments), gender, race/ethnicity and diabetes status. SSTI episodes were also summarized by year, age group, gender, race/ethnicity and diabetes status. Persons could contribute more than one episode to the numerators.

Incidence rates were calculated by dividing the number of episodes in each stratum by the person-years in that stratum. For reporting, one-year age strata were summarized into five age groups (less than 5, 5- < 18, 18- < 50, 50- < 65, and 65+ years of age). Ninety-five percent confidence intervals for incidence rates were calculated using the following formulas:

$$\begin{array}{l} \text{Lower}\;\text{confidence}\;\text{limit}\\ \;\;=\text{incidence}\;\text{rate}-1.96*\left(\sqrt{\text{episodes}}/\text{person}-\text{years}\right)\\ \text{Upper}\;\text{confidence}\;\text{limit}\\ \;\;=\text{incidence}\;\text{rate}+1.96*\left(\sqrt{\text{episodes}}/\text{person}-\text{years}\right) \end{array}$$

Rate ratios were estimated using a single multivariate negative binomial regression in which episodes from all years were included. Each record in the analytic dataset was one of the age, gender, race/ethnicity, and diabetes strata noted above. The dependent variable was the number of SSTI episodes for all patients in the stratum. Independent variables were gender, age group (less than 5, 5- < 18, 18- < 50, 50- < 65, and 65+ years of age), race/ethnicity, and diabetes status. The log of person-years in each stratum was used as an offset term. In a sensitivity analysis, we ran the same model but included only the first SSTI per patient.

### Predictors of S. aureus and MRSA among cultured SSTIs

We examined the relationship between demographic and clinical characteristics and the risk of *S*. *aureus* and MRSA. We created an analytic dataset that included all SSTI episodes from which a culture was obtained. Odds ratios were estimated using a multivariate logistic regression where the dependent variable was whether or not the culture was positive for *S*. *aureus* and the independent variables were gender, age (treated as a categorical variable with five levels: less than 5, 5- < 18, 18- < 50, 50- < 65, and 65+ years of age), race/ethnicity, diabetes status, quintile of census block income, and the type of SSTI. Because some patients had more than one SSTI between 2009 and 2011, we used a generalized estimating equation approach with an exchangeable covariance structure to account for correlation among records for the same person. The resulting odds ratios represent the odds of testing positive for *S*. *aureus* given that microbiological testing was performed. A second model was run to identify the risk factors for testing positive for MRSA among those SSTI episodes that were positive for *S*. *aureus*. The dependent variable in this model was whether or not the SSTI was positive for MRSA and the independent variables were the same as in the first model. The resulting odds ratios represent the odds of testing positive for MRSA given that the SSTI was culture-confirmed *S*. *aureus*.

## Results

### Incidence and predictors of clinically-diagnosed SSTIs

During the three-year study period, 376,262 unique individuals experienced 471,550 SSTI episodes (Table 1). The majority of these SSTIs were coded as “cellulitis and abscess” (63%), and the average age of patients was 41 years. The racial profile of SSTI patients was similar to that of the KPNC membership except that Asians were under-represented (12% of SSTIs versus about 18% of KPNC members) and Whites were over-represented (54% versus about 49%). Twenty-three percent of SSTI episodes were cultured, and 12% of all episodes had a potentially clinically-relevant pathogen identified.

**Table 1:** **Characteristics of clinically**-**diagnosed skin and soft tissue infection episodes**, **Kaiser Permanente of Northern California**, **2009**–**2011 **^**a**^

**All episodes**	**471,550 (100)**
Type of SSTI	
Carbuncle and furuncle	24,238 (5)
Impetigo	37,075 (8)
Other infections of the skin	31,870 (7)
Folliculitis	69,000 (15)
Other SSTI	11,331 (2)
Cellulitis and abscess	298,036 (63)
Gender	
Female	249,844 (53)
Male	221,706 (47)
Age in years	
Under 5	32,384 (7)
5- < 18	73,779 (16)
18- < 50	185,103 (39)
50- < 65	97,277 (21)
65+	83,007 (18)
Mean age (std)	40.82 (24)
Race/ethinicity	
Asian	55,595 (12)
African-American	43,407 (9)
Hispanic	96,626 (20)
Native American	2,967 (1)
Multiracial	18,449 (4)
White	254,507 (54)
Microbiology test performed^b^	108,243 (23)
Any pathogen identified by microbiology test	58,794 (12)

^a ^Episodes were defined as beginning on the date of the first skin and soft tissue infection (SSTI) diagnosis and ending with the last SSTI or SSTI-related complication diagnosis, not followed by another SSTI or SSTI complication diagnosis within 42 days. Duration of health services use was calculated as the number of days from the start of the episode to the end of the episode. Values in columns are number of episodes and percent of all episodes, unless otherwise indicated.

^b ^Microbiology tests included in analyses were those performed on blood, tissue, body fluid or other miscellaneous bacterial specimens (such as those taken from abscesses, pustules, boils, etc.) obtained from the patient within 7 days prior to the beginning of the SSTI episode to 7 days after the end of the episode. Respiratory, cerebrospinal fluid and urine specimens were excluded. These microbiology tests were considered to have been performed to determine SSTI etiology. A minority of episodes received such testing.

The rate of clinically-diagnosed SSTIs was 496 per 10,000 person-years, with the highest crude incidence rates being in persons 65 years of age and older, Native Americans, African-Americans, persons identified as Multiracial, and persons with diabetes (Table 2). After adjusting for patient demographics and diabetes (and including only main effects), children under 5 years of age had higher rates of SSTI than persons aged 65 years and older (Rate Ratio (RR): 1.21, Confidence Interval (CI): 1.17 to 1.26), while persons in other age groups had lower rates of SSTIs compared to persons aged 65 years and older. Asians (RR: 0.51, CI: 0.50 to 0.52), African-Americans (RR: 0.94, CI: 0.92 to 0.96), and Hispanics (RR:0.81, CI: 0.80 to 0.83 all had lower rates of SSTIs than Whites. Persons with diabetes had nearly double the rate of SSTI compared to persons without diabetes (RR: 1.93, CI: 1.90 to 1.96). When we restricted the model to include only the first episode per individual, the RR associated with diabetes decreased from 1.98 to 1.69 (CI: 1.66 to 1.72), but all other results remained substantially the same. When diabetes was excluded from the main model (the one allowing multiple episodes per person), the results were substantially the same as when diabetes was included, with the exception that children under 5 years of age had a lower RR (RR: 0.901, CI: 0.85 to 0.95) compared to persons aged 65 years and older, and Native Americans and persons identified as Multiracial join the other race/ethnicities in having lower risk of SSTI compared to Whites.

**Table 2:** **Incidence rates of clinically**-**diagnosed skin and soft tissue infection episodes and adjusted relative rate ratios**: **model with main effects only**

**Patient characteristic**	**Episodes per 10,000 person-years (95% CI)**	**Adjusted relative rate ratio (95% CI) **^**a**^
All	496 (494-497)	
Gender		
Female	506 (504-508)	1.05 (1.04,1.07)
Male	485 (483-487)	ref
Age (years)		
Under 5	579 (573-585)	1.21 (1.17,1.26)
5- < 18	440 (437-443)	0.88 (0.85,0.90)
18- < 50	462 (460-464)	0.98 (0.96,1.00)
50- < 65	488 (485-491)	0.87 (0.85,0.89)
65+	652 (648-657)	ref
Race/Ethnicity		
Asian	320 (317-322)	0.51 (0.50,0.52)
African-American	589 (584-595)	0.94 (0.92,0.96)
Hispanic	474 (471-477)	0.81 (0.80,0.83)
Native American	630 (608-653)	1.03 (0.99,1.07)
Multiracial	600 (591-609)	0.98 (0.96,1.00)
White	548 (546-550)	ref
Diabetes		
Yes	928 (921-936)	1.93 (1.90,1.96)
No	462 (461-464)	ref

Kaiser Permanente of Northern California, 2009 to 2011.

^a ^Rate ratios were derived from a single multivariate negative binomial model. The entire KPNC membership for years 2009-2011 were summarized into strata by gender, age, race, and diabetes status. Each record in the analytic dataset was one of these strata. The dependent variable was the number of SSTI episodes for all members in the strata. Independent variables were gender, age, race and diabetes status. The log of member years in each strata was used as an offset term. The model presented here includes only main effects, and thus represents averages across groups. Ref = reference group; CI = 95% confidence interval.

In a post-hoc analysis to investigate differences among the racial/ethnic groups in the relationship between age and the risk of SSTI, we re-ran the model but included an interaction term for race/ethnicity by age group. In this model, within each racial/ethnic group, the rates of SSTI in the younger age groups were compared to the rate in persons age 65 years and older (Table 3). This model indicated that children less than 5 years of age had a significantly higher rate of SSTI compared to persons 65 years of age and older in Asians (RR: 1.86, CI: 1.74 to 2.00), African-Americans (RR: 1.51, CI 1.40 to 1.62), and Hispanics (RR: 1.22, CI: 1.14 to 1.30), but not in Native Americans, persons classified as multiracial, or Whites. Also notable was that African-Americans aged 18 to <50 and 50 to <65 had substantially higher rates of SSTI compared to African-Americans 65 years of age and older (RR: 1.59, CI: 1.52 to 1.66, and, RR: 1.19, CI: 1.14 to 1.25, respectively). When the same model was rerun, but parameterized in such a way that within each age group the rate in Whites was the reference group, Asians were found, at every age, to have lower rates of SSTI than Whites of the same age, and Hispanics were found to have lower rates of SSTI at every age group except children under 5 years, compared to Whites of the same age group (not shown). African Americans aged 18 to <50 had a higher rate of SSTI (RR: 1.27, CI: 1.23 to 1.31) compared to whites of the same age, but at other ages had similar, or lower rates, than Whites.

**Table 3:** **Incidence rates of clinically**-**diagnosed skin and soft tissue infection episodes and adjusted relative rate ratios**: model including race*age interaction

**Patient characteristic**	Race * age interaction	**Adjusted relative rate ratio (95% CI) **^**a**^
All		
Gender		
Female		1.05 (1.04,1.07)
Male		REF
Diabetes		
Yes		1.95 (1.93,1.98)
No		REF
Race/ethnicity		
Asian		0.43 (0.41,0.44)
African-American		0.64 (0.61,0.67)
Hispanic		0.73 (0.71,0.75)
Native American		0.94 (0.85,1.04)
Multiracial		0.96 (0.92,1.00)
White		REF
Race/ethnicity * Age		
Asian	Under 5	1.86 (1.74,2.00)
	5- < 18	1.18 (1.12,1.24)
	18- < 50	1.05 (1.01,1.10)
	50- < 65	0.86 (0.82,0.90)
	65+	REF
African-American	Under 5	1.51 (1.40,1.62)
	5- < 18	0.99 (0.94,1.05)
	18- < 50	1.59 (1.52,1.66)
	50- < 65	1.19 (1.14,1.25)
	65+	REF
Hispanic	Under 5	1.22 (1.14,1.30)
	5- < 18	0.92 (0.88,0.96)
	18- < 50	0.96 (0.93,0.99)
	50- < 65	0.91 (0.87,0.94)
	65+	REF
Native American	Under 5	0.99 (0.82,1.20)
	5- < 18	0.81 (0.70,0.93)
	18- < 50	0.98 (0.87,1.10)
	50- < 65	0.95 (0.84,1.08)
	65+	REF
Multiracial	Under 5	0.94 (0.85,1.03)
	5- < 18	0.74 (0.69,0.78)
	18- < 50	0.85 (0.81,0.89)
	50- < 65	0.87 (0.83,0.91)
	65+	REF
White	Under 5	0.95 (0.89,1.00)
	5- < 18	0.78 (0.75,0.81)
	18- < 50	0.80 (0.78,0.82)
	50- < 65	0.78 (0.76,0.81)
	65+	REF

Kaiser Permanente of Northern California, 2009 to 2011

^a ^Rate ratios were derived from a single multivariate negative binomial model. The entire KPNC membership for years 2009–2011 were summarized into strata by gender, age, race, and diabetes status. Each record in the analytic dataset was one of these strata. The dependent variable was the number of SSTI episodes for all members in the strata. Independent variables were gender, race, race*age, and diabetes status. The log of member years in each strata was used as an offset term. Ref = reference group; CI = 95% confidence interval.

### Rates and microbiology of SSTIs with microbiologic testing

Among episodes with a culture, 54% had a pathogen identified (Table 4). *S*. *aureus* was by far the most common pathogen, isolated in 81% of pathogen-positive index specimens. Among *S*. *aureus* isolates, 46% were MRSA (37% of all pathogen-positive index specimens). Other important pathogens were BHS (10% of pathogen-positive index specimens) and gram negative bacteria (14%). Multiple pathogens were identified in 6% of index specimens with a pathogen.

**Table 4:** **Skin and soft tissue infections with microbiologic testing**, **Kaiser Permanente of Northern California**, **2009**–**2011 **^**a**^

**SSTI episodes with microbiologic testing**	**108,243 (100)**
Any pathogen identified by micriobiology test	58,794 (54)
Specimen type^b^	
Blood	14,761 (14)
Other	93,482 (86)
Microbiology results: Among episodes with culture-confirmed pathogen^c^	
MRSA	21,890 (37)
MSSA	25,628 (44)
BHS	5,729 (10)
Other streptococci	72 (<1)
Other gram positive bacteria	923 (2)
Gram negative bacteria	7,955 (14)
Anaerobic bacteria	101 (<1)

^a ^Only SSTIs with a microbiology test are included in this table. Only tests performed on specimens obtained in the period from 7 days prior to the start of the SSTI episode to 7 days after the end of the SSTI episode were included. These tests were considered to have been performed to determine SSTI etiology. MRSA = methicillin-resistant *S*. *aureus*; MSSA = methicillin-sensitive *S*. *aureus*; BHS = beta-hemolytic streptococcus. Data are number (%) of cultured SSTI episodes, unless otherwise indicated.

^b ^Specimen type was the first positive specimen during the episode, or, if no specimens were positive, the first specimen. “Other” specimens include tissue, body fluid, and other miscellaneous bacterial specimens such as those taken from abscesses, pustules, boils, etc. Respiratory, CSF and urine cultures were excluded.

^c ^Denominator for percents is the number of episodes with any positive pathogen. Pathogen results are not mutually-exclusive. Over the study period 6% of first positive specimens were positive for multiple organisms. “Other streptococci” comprises *S*. *pneumoniae*, *S*. *milleri*, *S*. *anginosus*, *S*. *constellatus*, and *S*. *intermedius*. The following organisms were considered contaminants and not included: coagulase negative staphylococcus, *ochrobactrum*, *corynebacterium*, *micrococcus spp*., *bacillus spp*., gram positive cocci, gram positive rods, and streptococcus species other than BHS, *S*. *pneumoniae*, and “other streptococci” as defined above. ”BHS” comprises streptococci with Lancefield groups A, B, C and G.

### Predictors of S. aureus and MRSA

Among SSTI episodes with a culture, there was a consistent trend of younger persons being more likely to test positive for *S*. *aureus* (Table 5). Episodes in children under 5 years of age had 3.89 (CI: 3.64 to 4.14) times the odds of being *S*. *aureus* compared to episodes in persons 65 years and older. Hispanics were somewhat more likely to test positive for *S*. *aureus* than were whites, and persons living in less affluent census blocks were more likely to test positive for *S*. *aureus* than persons in more affluent census blocks. Carbuncle/furuncle and cellulitis and abscess were more likely to be culture-positive for *S*. *aureus* than were other types of SSTIs.

**Table 5:** **Risk factors for *S***. ***aureus *and methicillin**-**resistant *S***. ***aureus *skin and soft tissue infection episodes**, **Kaiser Permanente of Northern California**, **2009**-**2011**^**a**^

	**Adjusted odds ratio (95% CI)**^**b**^	
	**Among all episodes with a culture (n = 108243)**^**c**^	**Among episodes positive for *S*. *aureus *(n = 47518)**^**d**^
**Characteristic**	**Odds of *S*. *aureus *vs. not *S*. *aureus***	**Odds of MRSA vs. MSSA**
Gender		
Female	0.86 (0.84,0.88)	1.04 (1.00,1.09)
Male	ref	ref
Age (years)		
Under 5	3.89 (3.64,4.14)	1.28 (1.17,1.40)
5- < 18	2.64 (2.52,2.78)	0.79 (0.73,0.85)
18- < 50	1.91 (1.83,1.98)	1.11 (1.04,1.18)
50- < 65	1.40 (1.34,1.46)	1.03 (0.96,1.10)
65+	ref	ref
Race/Ethnicity		
Asian	1.02 (0.98,1.06)	0.73 (0.68,0.78)
African-American	1.02 (0.98,1.07)	1.79 (1.67,1.92)
Hispanic	1.05 (1.01,1.08)	1.24 (1.18,1.31)
Native American	1.11 (0.95,1.30)	1.16 (0.92,1.46)
Multiracial	0.95 (0.89,1.02)	1.01 (0.91,1.13)
White	ref	ref
Diabetes status		
Diabetes	0.93 (0.90,0.97)	0.75 (0.71,0.80)
No diabetes	ref	ref
Census Block Income Quintile		
Unknown	1.16 (1.10,1.23)	1.27 (1.17,1.38)
1st (lowest income)	1.17 (1.12,1.22)	1.42 (1.33,1.51)
2nd	1.13 (1.09,1.18)	1.25 (1.17,1.34)
3rd	1.08 (1.03,1.12)	1.15 (1.08,1.22)
4th	1.02 (0.98,1.07)	1.12 (1.05,1.20)
5th (highest income)	ref	ref
Type of SSTI		
Carbuncle and furuncle	1.33 (1.26,1.40)	1.22 (1.14,1.30)
Impetigo	1.03 (0.97,1.09)	0.16 (0.14,0.18)
Other infections of the skin	1.06 (1.00,1.11)	0.55 (0.51,0.59)
Folliculitis	0.87 (0.83,0.91)	0.50 (0.46,0.53)
Other SSTI	0.48 (0.42,0.53)	0.68 (0.56,0.82)
Cellulitis and abscess	ref	ref

^a ^CI = 95% confidence interval; MSSA = methicillin-sensitive *S*. *aureus*; MRSA = methicillin-resistant *S*. *aureus*; ref = reference group for other odds ratios within characteristic.

^b^Odds ratios estimated using multivariate logistic regression. A separate model was run for each column in the table. For both models the independent variables were gender, age, race/ethnicity, diabetes status, income quintile based on census block, and type of SSTI.

^c ^The analytic dataset had one record per SSTI episode with a microbiologic test, and the dependent variable was whether or not the test was positive for *S*. *aureus*. Specimens may have been positive for other organisms or no organism may have been identified. Episodes without a microbiologic test were not included in the model.

^d ^The analytic dataset had a record for each SSTI episode that tested positive for *S*. *aureus*. The dependent variable was whether or not the isolate was methicillin-resistant.

Among SSTI episodes with a positive *S*. *aureus* culture, isolates from females were slightly more likely to be MRSA (Odds Ratio (OR): 1.04, CI: 1.00 to 1.09, compared to males). Compared to Whites, isolates from African-Americans were more likely to be MRSA (OR: 1.79, CI: 1.67 to 1.92) as were those of Hispanics (OR: 1.24, CI: 1.18 to 1.31). Isolates from Asians, on the other hand, were substantially less likely to be MRSA than those of whites (OR: 0.73, CI: 0.68 to 0.78) or other racial/ethnic groups. Isolates from persons living in less affluent census blocks were more likely to be MRSA compared to isolates from persons living in more affluent census blocks. Isolates from diabetics were less likely to be MRSA than isolates from non-diabetics (OR: 0.75, CI: 0.71 to 0.80), and carbuncle/furuncle and cellulitis and abscess isolates were more likely to be MRSA than were isolates from other types of SSTI (OR: 1.22, CI: 1.14 to 1.30).

## Discussion

Over the three-year period from 2009 to 2011, we found that the incidence of clinically-diagnosed SSTIs in this population was nearly 500 episodes per 10,000 person-years. By contrast, this rate is more than double the incidence of clinically-diagnosed pneumonia (approximately 190 per 10,000) in this population (unpublished data), although pneumonia is more likely to result in hospitalization.

In one of the few U.S. population-based studies of the incidence of community-onset SSTIs published in the last decade, Simonsen et al., using 1997 to 2002 data from a Western U.S. medical insurance claims database, reported the incidence rate of cellulitis and abscess in persons less than 65 years of age to be 241 per 10,000 person years [31]. The corresponding rate in our population for 2009–2011 (when restricting the analysis to cellulitis and abscess in persons under 65 years of age) was essentially the same (about 244 per 10,000 person-years).

In the multivariate model adjusting for gender, age group, race/ethnicity, and diabetes status, being under 5 years of age, and having diabetes were the two largest risk factors for being diagnosed with an SSTI. The increased risk of SSTIs among diabetics may, in part, be related to increased opportunities to get diagnosed, variations in care-seeking behavior, and other comorbidities, rather than directly related to diabetes [44]. However, one study that adjusted for some of these factors also found that diabetics were at increased risk of skin infections [35]. White race was also a risk factor for being diagnosed with SSTI. In a post-hoc analysis, we found that the relationship of age to the rate of clinically-diagnosed SSTI was different depending on race/ethnicity. Notably, unlike whites, the highest rate of SSTI in Asians, Hispanics, and Native Americans was in the youngest age group, rather than the elderly. Among African-Americans, although the youngest age group also had a higher rate of SSTI than the elderly, persons 18- < 50 years of age had the highest rate of SSTI. That the effect of age group was significantly different by race/ethnicity, reminds us that models that include only main effects may mask important differences.

Because of inability to determine microbiologic etiology of many SSTIs, and potential bias with respect to which SSTIs get cultured, it is difficult to infer the true underlying proportion of SSTIs due to given pathogens. The sample of SSTIs that get cultured are not representative of all SSTIs, especially given that many SSTIs are typically not culturable (e.g., cellulitis without abscess). In our setting, 23% of SSTI episodes were cultured, and a potentially clinically-relevant pathogen was isolated in 54% of those episodes. Over 80% of pathogen-positive specimens were *S*. *aureus* while only 10% were BHS. However, studies indicate that non-culturable cellulitis may be more likely to be caused by BHS than *S*. *aureus *[6], so BHS may represent a more important cause of SSTIs than the results of cultures indicate.

Among *S*. *aureus* isolates, we found that 46% were MRSA. Prior studies have shown that *S*. *aureus* is the leading cause of cultured SSTIs [7-11,45-50], and that the percent of these that were MRSA increased during the early 2000’s [7,51,52]. However, most of these studies only included hospitalized cases [7-11,51] or were for special populations (i.e., children [51] and the uninsured [49,50]). In a 2004 study of specimens (n = 422) taken from ambulatory patients with SSTIs (n = 422), Moran et al. reported that *S*. *aureus* was isolated in 76% of the specimens, and 78% of these were MRSA [47]. MRSA may have been more prevalent in the Moran et al. population compared to KPNC in part because of the larger percent of African-Americans and Hispanics in that population, which, in the KPNC population, were more likely to have *S*. *aureus* SSTIs that were MRSA than Whites or Asians. In a study of San Francisco Bay Area outpatients (n = 529) presenting with purulent or fluid-producing SSTIs during 2006–2007, Weiss et al. found that, among those with microbiologic testing, 22% of specimens were positive for MRSA and 30% for methicillin-sensitive *S*. *aureus* (MSSA) [45]. These compare to 20% (MRSA) and 24% (MSSA) in our study, when the denominator was all SSTIs with microbiologic testing.

While diabetes was associated with higher risk of SSTIs (and higher absolute rates of MRSA), it was not associated with an increased risk of MRSA infection compared to MSSA – in fact, it was associated with a decreased risk. This finding is consistent with a recent study of hospitalized patients with complicated SSTIs and a positive blood or skin culture, which found that not having evidence of diabetes corresponded to an increased likelihood of the culture being MRSA [53]. In part, these findings in our population may be related to a higher propensity of providers to order cultures for diabetics. We found that 31% of SSTI episodes for diabetics were cultured, while only 22% of SSTI episodes for non-diabetics were cultured, and that the cultures obtained from diabetics were more likely to be blood than were the cultures from non-diabetics (24% vs. 11%). Thus, for diabetics, providers may have disproportionately ordered cultures for types of SSTIs that were less likely to MRSA. These findings related to diabetics should be further explored.

Consistent with a number of prior studies [12,14,27,54-56], we found that, among persons with *S*. *aureus* infections, African-Americans and persons living in lower-income census blocks were at substantially increased risk of their *S*. *aureus* being methicillin-resistant. We also found that Asians were at substantially lower risk of being diagnosed with an SSTI, and that their SSTI-related *S*. *aureus* isolates were substantially less likely to be methicillin-resistant than those of other racial/ethnic groups. No US population-based study has, to our knowledge, previously reported on rates of SSTI and likelihood of *S*. *aureus* being methicillin-resistant in persons of Asian race vis-à-vis persons of other racial/ethnic groups. However, Liu et al. reported that, among San Francisco residents, Asian/Pacific Islanders had the lowest rates of community-onset and hospital-onset MRSA disease, much of which was likely related to SSTI [54]. Our findings with respect to Asians are novel and should be confirmed in other populations.

### Limitations

Our selection criteria explicitly excluded SSTIs diagnoses that we thought likely be represent nosocomial or chronic infections, and thus our incidence rates and microbiology results do not reflect inclusion of those types of infections. In addition, our findings reflect the routine practice of providers in this health plan with respect to diagnosing and microbiologic testing, and these practices may differ from those of other settings. The differences we found in risk of SSTIs among the different gender, race/ethnicity, and age groups, may to some extent reflect differences in the propensity of persons in these groups to seek treatment. As an observational study, our analysis of risk factors could be affected by unmeasured confounders. We did not include, for example, nursing home status, family size, injection drug use, or medical conditions other than diabetes. We included diabetes as a risk factor due to the high prevalence of the condition, and because prior literature suggests its relationship to increased risk of SSTI [35-37,39,40]. However, other conditions – in particular, immuno-compromising conditions such as HIV – might also be associated with increased risk of SSTIs. In this study we did not assess severity or health services utilization. Therefore, higher risk of clinically-diagnosed SSTIs does not necessarily imply more severity or greater overall cost of care. Cultures were included based on their temporal proximity to a patient’s SSTI diagnosis. It is possible that some patients may have multiple infections, and that the pathogen we associated with the SSTI infection may not have been the cause of that infection.

## Conclusions

SSTIs represent a significant burden to the health care system. The majority of culture-positive SSTIs were caused by *S*. *aureus*, and half of the *S*. *aureus* SSTIs were methicillin-resistant. After adjusting for demographic variables and diabetes status, diabetics, persons in the lower economic strata, and children under 5 years of age were at particular risk of SSTI, while Asians were at lower risk. Our findings that the relationship of age to the rate of SSTIs differed considerably by race/ethnicity warrants further investigation.

## Abbreviations

SSTI: Skin and soft-tissue infection; S. aureus: *Staphylococcus aureus*; BHS: Beta-hemolytic streptococci; MRSA: Methicillin-resistant *Staphylococcus aureus*; MSSA: Methicillin-sensitive *Staphylococcus aureus*; KPNC: Kaiser Permanente of Northern California; CA-MRSA: Community-associated methicillin-resistant *Staphylococcus aureus*; ICD9-CM: international classification of disease, 9th revision, clinical modification; OR: Odds ratio; CI: Confidence Interval.

## Competing interests

G. Thomas Ray has received research support from GlaxoSmithKline, Pfizer, Merck and Purdue Pharma L.P. Roger Baxter has received research funding from GlaxoSmithKline, Pfizer, Merck, Sanofi Pasteur and Novartis. Jose A. Suaya is an employee of GlaxoSmithKline and received restricted shares of company stock.

## Authors’ contributions

GTR contributed to the design of the study, made substantial contributions to the data acquisition, data analysis, and interpretation of data, and was primarily responsible for drafting the manuscript. JAS contributed to the design of the study, made substantial contributions to the interpretation of data, and helped to draft the manuscript and revise it for important intellectual content. RB conceived the study, contributed to the design of the study, made substantial contributions to the interpretation of data, and helped to draft the manuscript and revise it for important intellectual content. All authors read and approved the final manuscript. The authors thank Heather Santiago (GlaxoSmithKline Vaccines) for managing the manuscript.

## Pre-publication history

The pre-publication history for this paper can be accessed here:

http://www.biomedcentral.com/1471-2334/13/252/prepub
